# Personalizing computational models to construct medical digital twins

**DOI:** 10.1098/rsif.2025.0055

**Published:** 2025-07-02

**Authors:** Adam Knapp, Daniel A. Cruz, Borna Mehrad, Reinhard C. Laubenbacher

**Affiliations:** ^1^ Department of Medicine, University of Florida, Gainesville, FL, USA

**Keywords:** medical digital twin, agent-based model, data assimilation, ensemble Kalman filter

## Abstract

Digital twin technology, originally developed for engineering, is being adapted to biomedicine and healthcare. A key challenge in this process is dynamically calibrating computational models to individual patients using data collected over time. This calibration is vital for improving model-based predictions and enabling personalized medicine. Biomedical models are often complex, incorporating multiple scales of biology and both stochastic and spatially heterogeneous elements. Agent-based models, which simulate autonomous agents, such as cells, are commonly used to capture how local interactions affect system-level behaviour. However, no standard personalization methods exist for these models. The main challenge is bridging the gap between clinically measurable macrostates (e.g. blood pressure and heart rate) and the detailed microstate data (e.g. cellular processes) needed to run the model. In this article, we propose an algorithm that applies the ensemble Kalman filter, a classic data-assimilation technique, at the macrostate level. We then link the Kalman update at the macrostate to corresponding updates at the microstate level, ensuring that the resulting microstates are compatible with the desired macrostates and consistent with the model’s dynamics. This approach improves the personalization of complex biomedical models and enhances model-based forecasts for individual patients.

## 1. Introduction

A medical digital twin (MDT) is a virtual replica of some aspect of a patient’s biology relevant to their health; it consists of a computational model that is calibrated to the patient and is dynamically updated from data repeatedly collected from the patient. The MDT can be used to forecast a patient’s health trajectory or simulate the effect of different interventions. MDTs promise to be a key technology for the implementation of personalized medicine. Preliminary work associated with MDT implementation and architecture (e.g. how MDTs and their associated data will be stored, accessed, etc. in clinical settings) is already underway [1–4], stemming from the use of digital twins in engineering. While there are some examples that deliver on the promise of MDTs, such as the artificial pancreas [5], several foundational problems need to be solved first [6–8] before this technology can be used widely.

In this article, we address the core mathematical problem of calibrating a generic computational model to a particular patient, forecasting disease progression and the effect of therapeutic interventions. We study data assimilation (DA), the integration of observational data into a computational model, for the purpose of personalizing the model. Specifically, we consider how to find likely states and parametrizations of the model so that we can improve the prediction of a patient’s health outcomes. Finding the precise state and parameters of a patient, especially given small amounts of available data, is a challenging task: models may have structurally or practically unidentifiable parameters in this regime [9], and many model states and parameters may reproduce the observed data. To tackle this challenge, we use parameter and state distributions that capture these uncertainties.

One of the features that sets applications of DT technology to human health apart from other areas, such as numerical weather prediction or most industrial applications, is that there are different model types for different aspects of human biology. In particular, many biological features are characterized by spatial heterogeneity, such as lung biology, or by their inherently stochastic nature, such as the immune system. This makes it often infeasible to use the framework of ordinary differential equations, for which many mathematical tools are available. An increasingly important modelling framework in this context are agent-based models (ABMs), a mechanistic modelling framework which is common in cellular biology [10–13] and biomedicine, with applications ranging from respiratory diseases to immune-mediated diseases and cancer [14–20], and is supported by a growing list of software platforms [21–24]. For instance, elements of the immune response to pathogens can be effectively captured with this framework by taking advantage of spatial heterogeneity to properly capture the effect of small numbers of cells in certain regions, such as resident macrophages in lung alveoli.

Detailed mechanistic models are capable of reproducing a variety of patient outcomes (see [5,25,26]) and are motivated by the need to optimize mechanism-based interventions. A drawback is that, in addition to clinically measurable quantities, they often include practically unobservable quantities. These mostly relate to the basic feature of ABMs that the individual agents (e.g. cells) have internal states, spatial positions and interact with each other and the spatial environment, generating what we refer to as ‘microstates’, which contain the fine-grained information required to compute the model. In contrast, measurements taken from a patient that can be used to personalize a generic mechanistic model to the patient, typically capture ‘macrostates’—aggregate measures such as blood cytokine levels or lung immune cell counts. The interplay between micro- and macrostates lies at the heart of the DA challenges for ABMs, and is the topic of the current paper.

Extensive work has been done on applying DA techniques to such ABMs [27–29], especially in the context of transportation systems [30–40] and social/epidemiological spread [41–47]; however, little work has been done related to biomedical applications. Moreover, the existing research generally falls into two categories: (i) DA done using full microstates of ABMs and (ii) DA done on surrogate models built from an ABM. For the former, considering the full microstate of an ABM will typically include granular details, which are not medically relevant, will likely rely on data that may not be available and may take on a form which is incompatible with standard techniques. The high-dimensionality of such a system requires a very large ensemble to give adequate estimates of many state variables, especially when taking a Bayesian learning approach, resulting in high computational costs inappropriate to the better-than-real-time requirements of an MDT. For the latter, MDTs will require the inclusion of mechanistic controls in the underlying model to allow physicians to evaluate the effects of therapies. Designing surrogate models that include such mechanistic controls is a challenging task in its own right which has not been resolved generally [48,49]. However, future applications may require these issues to be resolved, as surrogate models may be required in the case that the ABM runs slower than real time.

Our main methodological contribution addresses the following mathematical problem: given an ABM and repeated partial measurements of macrostates, how can we recalibrate the ABM and simulate it to make a prediction about its likely trajectory? To address this question, we develop a hybrid method (in the form of two algorithms), which uses an ensemble Kalman filter method [50] on the comparatively low-dimensional macrostates and then updates the microstates, which are used to generate new ABM predictions. The first algorithm performs a Kalman update, which modifies model macrostates with partial measurements as they become available; however, these macrostates need to be translated to equivalent microstate modifications. The second algorithm and main technical contribution of this article is a microstate synthesis algorithm that generates ‘likely’ microstates associated to a given macrostate.

To explore the utility and performance of our algorithms, we apply our methodology to two example ABMs. We evaluate the performance of our algorithm using ‘surprisal’, a concept which we introduce in the following section and define formally in the electronic supplementary material. The first ABM is a spatially well-mixed model of predator–prey dynamics [51] for which the microstate synthesis problem is relatively simple. For this model, we get good performance of the methodology overall. However, we find that there are certain cases that the present algorithm cannot handle. These occur for certain absorbing states of the model that cannot be distinguished by the observables at hand. The second ABM is a model of viral dynamics [52], which exhibits a number of more complex features in the microstate, including a high degree of spatial heterogeneity. To deal with this increase in complexity, we apply a microstate synthesis algorithm which employs quantization and error diffusion. For this model, our analysis uncovered the existence of several model phenotypes and we explored the performance of the algorithm at the point where these phenotypes diverge.

## 2. Methods

The process of integrating patient data into a computational model is an example of DA; the study of how to optimally combine observed data and theory, in the form of a mathematical or computational model, into a predictive forecast. In order to provide a concrete example of the type of eventual applications that we envision for our algorithm, we describe a scenario below in an intensive care unit setting where a doctor might benefit from a decision support tool. Such a tool would provide forecasts of the patient’s health trajectory, based on mechanistic simulation and possible interventions available to the doctor. Another concrete example of how a DT could be applied to a clinical setting, specifically in the context of tumour growth in a cancer patient, can be found in [7]. We also point the reader to two publications that contain a number of concrete examples of digital twin use cases, with projects at different stages of completion [53,54]. These potential use cases have not yet been realized as working examples since there are many foundational problems to be solved before this technology can be applied broadly, as noted in the National Academies report [7]. This article is intended to address one of these foundational problems.

**Remark 1.** *We present the case of a patient cared for by one of the authors B.M. as a prototypical scenario to which simulation-based decision support algorithms could be applied. The patient was a 41 year old man with class I obesity (body mass index 34) and no other medical history who presented to the emergency department in November 2021 after a 9 day history of fever, cough, myalgia and progressive shortness of breath. On presentation, he was febrile, had normal blood pressure and had an oxygen saturation on room air was 85%*. *His chest X-ray showed bilateral airspace disease, and he tested positive for COVID-19. He required endotracheal intubation and mechanical ventilation for acute respiratory distress syndrome, and was treated with **remdesivir and dexamethasone**. Over the next 12 days, he developed worsening ARDS, septic shock requiring escalating doses of vasopressors and acute kidney injury. He died on the 13th hospital day of refractory shock*.

In the context of an MDT, one of our principal goals is to determine what kinds of interventions can be applied to a patient like the one in Remark 1 to improve outcomes. This requires that the model is sufficiently well calibrated or personalized to the patient to give accurate predictions of trajectories and the effects of interventions. However, realistic mechanistic models typically have high-dimensional and complex states, far exceeding the amount of data that can be expected from clinically feasible patient measurements. After model construction, the next task in building an MDT is to develop techniques to solve the ill-posed inverse problem of estimating the patient’s current state from the available data.

Typical methods for solving ill-posed inverse problems include forms of regularized maximum likelihood optimization, such as Tikhonov regularization, or are based on Bayesian statistical methods. It is not clear how regularization alone could provide a quantification of uncertainty into its forecasts, a key component of MDTs [7]. For this reason, we have opted to use a Bayesian filtering technique to predict patient outcomes, inspired by Kalman filter (KF) methods used in numerical weather forecasting [55–61]. During review, we were made aware of the related ‘equation-free’ method [62–65], which was primarily developed for differential equations models and uses a similar two-level macrostate/microstate scheme to the one we introduce in §3. Notable differences between our method and the equation-free method appear in microstate synthesis (in the equation-free terminology ‘lifting’): the equation-free method uses a deterministic lifting map while we sample from a conditional distribution.

Bayesian frameworks have several advantages, including a natural means of using prior knowledge of reasonable initial conditions and parameter values from a reference population. The use of an informative prior is an important technique to deal with the large number of parameters in complex models and paucity of data that can be collected clinically, especially early in treatment. Bayesian techniques also provide a convenient context with which to integrate uncertainty into forecasts. In particular, KF methods have several moving parts which are described statistically: (i) a predictive model, (ii) a true/target state, and (iii) observations/measurements. These methods have two steps:

(1) Predict: Given an initial distribution for states at time $t_{n}$ , the model is used to produce predictive state distributions for times $T>t_{n}$ .
(2) Update: When a measurement is taken at time $t_{n+1}>t_{n}$ , the predictive distribution at time $t_{n+1}$ is updated based on the measurement and predictive distribution at $t_{n+1}$ .

The updated distribution at time $t_{n+1}$ is then fed back into the predictive step as the initial distribution of states at time $t_{n+1}$ and the process begins again. An initial state distribution at t=0 is required to start the process. It is worth noting that, with current hardware, the full non-parametric Bayesian approach in a high-dimensional space (e.g. an ABM microstate) is too computationally intensive to satisfy the real-time requirements of an MDT and simplifying assumptions will need to be introduced. KF methods significantly reduce the computational load of the Bayesian approach by making a Gaussian assumption on distributions; however, these methods are difficult to apply directly to ABMs, as we mention in the Introduction and discuss in §3.

The KF [66–68] is the closed-form solution to the Bayesian filtering equations in the case where the dynamic and measurement models are linear stochastic and all distributions are (multivariate) Gaussian. We discuss the filter in more detail in the electronic supplementary material; however, we note that there have been numerous extensions of the KF including the extended Kalman filter (EKF) [69,70], ensemble Kalman filter (EnKF) [50,71] and unscented Kalman filter (UnScKF) [72], each of which contains tunable hyperparameters related to measurement and process uncertainty. The KF has already used in some preliminary MDTs based on ordinary and partial differential equations [2,4]. In this work, we use the EnKF because it tends to be most suitable for nonlinear stochastic models and is at the core of several numerical weather prediction models [56,57,59–61,71]. The linear stochastic dynamic model of the KF is replaced by a general computational model of dynamics in the EnKF, and the predictive distributions $x_{k}∼N({\hat{m}}_{k},{\hat{P}}_{k})$ are computed by selecting an *ensemble* of samples from $N(m_{k-1},P_{k-1})$ *,* running the computational model on each to obtain an ensemble of samples for the predictive distribution.

An appropriate measure is necessary to evaluate KF performance. Average or relative distances between ensemble mean predictions and the corresponding true trajectory do not account for predictive uncertainty. We chose a concept from information theory called *surprisal* [73], which takes these uncertainties into account by measuring the amount of information gained from revealing the true trajectory given a predictive distribution; see the electronic supplementary material for definitions. Informally, surprisal indicates how surprised we should be to learn the true trajectory; thus, high probability events will have low surprisal and vice versa. Finally, the KF includes hyper-parameters related to measurement uncertainty and process uncertainty which need to be adapted to the disease model at hand.

## 3. Results

### 3.1. The Kalman filter on agent-based models

**Figure 1.**
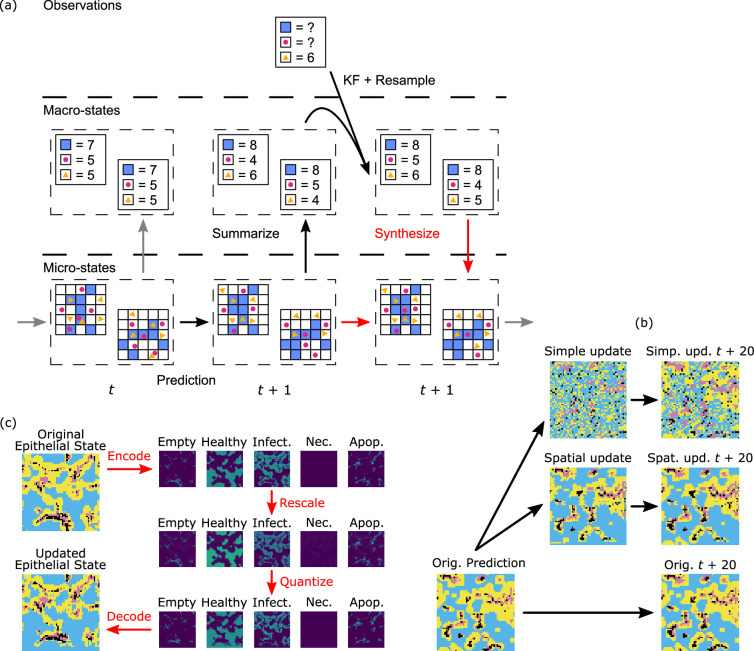
(a) Overview of the ABM Kalman filter (ABMKF) algorithm's update of microstates based on updates to the macrostates. Prediction: an ensemble of microstates at time t is advanced to t+1 using the mechanistic model. Summarize: the updated microstates are then summarized into macrostates. Resulting macrostates are used as the ensemble in the EnKF. Update: the EnKF resamples the updated ensemble of macrostates. Synthesize (in red): the previous microstates are used as seeds for the synthesis of new macrostate-compatible microstates. (b) Starting epithelial microstate (time t ). Second column: microstate updated by the two synthesis algorithms. Third column: microstates 20 time steps in the future. (c) Categorical epithelial states are encoded into five component scalar states as indicator variables. This ‘one-hot encoding’ [74] is rescaled to increase the healthy and decrease the infected component sums. The scaled field is quantized back to a one-hot encoding using the quantization/error-diffusion algorithm. Finally, the quantized one-hot encoding is decoded as a categorical state.

In order to make use of KF methods in ABM (or other) with complex state spaces, we will need to take care to define several of the mathematical objects considered in the sketch above. First, it is useful to consider which state components to include, not limiting ourselves to quantities for whom the model includes dynamics, but also including quantities that are often thought of as model parameters, as long as they vary between individuals and have a meaningful impact on model dynamics. As discussed below, it is often useful to expand the model to include random walk dynamics on these quantities to improve KF performance. Furthermore, the complex state spaces of ABMs lead to several complications, which lead us to a distinction between fully described microstates and more summarized macrostates upon which the KF acts (see figure 1a).

While we will not attempt to provide a formal definition of an ABM, we note that many computational models that go by this name have some commonalities [12,13,75–77]. Such a model contains one or more types of agent. For example, in the Wolf-Sheep-Grass (WSG) model considered later in §3, wolves and sheep are mobile agents and each grass patch is an immobile agent. An ABM of the immune system might contain several agent types, one for each type of immune cell considered as well as epithelial and endothelial cells, such as in the viral dynamics model discussed in later in §3. Such an ABM will typically support multiple instances of each agent type, each of which has its own state described by the same collection of data and updated by the same computational model. Often, these agents may enter or leave the simulation through a birth/death process.

These features lead to problems with applying KF methods directly to such a model:

(1) The space of states for the ABM may be broken into components which vary in dimension. The birth/death process for agents changes the total amount of data required to completely describe the model state, leading to dimensional changes when the number of agents change. This means that an ensemble of model instances may have members whose states do not live in the same dimension.
(2) There is often no natural matching of agents between ensemble members. This means that, even in the absence of a birth/death process, there is no *a priori* way of identifying the state spaces of model instances.
(3) An agent’s state may not be a vector space. For example, an ABM may drive agent behaviour through a Boolean network or a categorical state, leading to a (partially) discrete state space. Since the KF naturally produces continuous vector-valued states, models would not be able to use these directly and require the development of discretization techniques.
(4) Many ABMs include spatial components leading to excessively high dimensionality: a molecular density field might correspond to a variable taking values over an N×M lattice. That is, it corresponds to N×M dimensions in the global state space. Given K such molecules, we have at least KMN dimensions in the global state space and the covariance matrices used in the KF would then have up to $\frac{1}{2}KNM(KNM-1)$ entries to estimate. For example, in the viral dynamics model considered below we have K=12,M=N=51 so that we are discussing the estimation of over 487 million values in the covariance matrix. Given that the likely observables of such fields are merely the K spatial averages, this is a lot of computational cost to bear.

A possible solution to both problems is suggested by considering analogous problems in statistical mechanics and kinetics. In statistical mechanics, one makes a distinction between microstates and macrostates. A *microstate* is a complete description of a system’s state. For a gas, this might be a list of the position and momentum of all its constituent particles. In this setting, a *macrostate* is a description of the gas as a whole such as pressure, volume, number of molecules and temperature.

We intend to perform DA using KF methods on macrostates, as implied in [45] and fully proposed in [46]. However, there is an immediate problem: our models require the full microstate to run, and while the KF gives us a method of updating the distribution of model macrostates after an observation, it does not immediately lead to methods whereby we can update the corresponding distribution of microstates.

In our context, we propose some possible components to define the macrostate of the model so that it is useful for KF methods:

— For a collection of N agents, a quantity that is invariant under agent permutation; e.g. in a gas, the sum of the momenta. Averages and (co)variances of agent properties.
— The number of agents of a given type, especially when that number is relatively large.
— Average or total values of spatially distributed quantities. Small neighbourhood statistics.
— Realistically measurable quantities. For MDTs, this may include measurement of various substances measured in blood but exclude measurements with detailed spatial information about the distribution of these substances in an organ.

These quantities have some desirable qualities, such as being relatively stable over short times, frequently more compatible with realistic measurements and being approximately continuous.

While the summarization map from microstates to macrostates is a straightforward computation, the map is many-to-one. This means that, given a macrostate, there are many possible microstates representing it and the *microstate synthesis* step in figure 1a requires some choices. In the statistical mechanics analogy, we know that for a given macrostate ( P , T , n , V ), there are many possible ways of arranging the n particles in three-space. In statistical mechanics, a fundamental assumption is that each of these configurations is equally likely. This assumption may or may not be true for any given agent type in an ABM and we will see examples of both below. When the equiprobable assumption is true, there is a clear algorithm to synthesize microstates: generate a random microstate compatible with the macrostate through some uniform procedure. This could be generalized to the case where there is an explicit expression of microstate distributions for each macrostate, but it is very common that such an expression will be unavailable. We describe one technique for this case of microstate synthesis in §3.2.

The purpose of the MDT will determine a minimum of which components must included in the macrostate. In principle, all likely clinical measurements which could be derived from the model and its microstates could be included in the macrostate. However, these come at a cost, as the number of samples required to accurately estimate the means and covariances in the KF depends on the overall dimension of the macrostate space. We select the macrovariables based on a combination of measures reported in the source articles and expert input as to what could be clinically measurable. An application of this method should consider trade-offs between the information gained by including a macrovariable and the additional computational cost. Beyond the inclusion or omission of a macrovariable, we must also consider variable transformations (see the electronic supplementary material for more details).

### 3.2. Microstate synthesis for biological agent-based models

Extending KF-based methods to ABMs requires a microstate synthesis algorithm. We propose a general principle for microstate synthesis algorithms for EnKFs and specific implementations for the models we present as use cases. Constructing such an algorithm has been straightforward in prior work [45,46] because of the presence of discrete model compartments, which house agents which correspond to a comparatively small difference between microstates and macrostates; these compartments allow for the macrostate to be composed of only agent counts per compartment. However, the presence of such compartments is not guaranteed in general, especially in ABMs which feature spatial attributes.

The first step for our algorithm is initialization of the ensemble. These initial microstates will depend on parameters sampled from a Gaussian prior distribution on macrostates. For the examples of interest here, it is comparatively straightforward to create the details of the corresponding initial microstates as they will be in a known configuration; e.g. in the patient described in the introduction, an estimate of their state at the onset of infection. This initialization gives us an initial micro- and macrostate ensemble.

The prediction/correction, which we call the ABMKF, algorithm is then:

(1) Advance the microstate ensemble using the model, producing samples from a predictive microstate distribution.
(2) Summarize predictive microstate trajectories to predictive macrostate trajectories.
(3) Using a macrostate-derived measurement, perform ensemble KF methods to the predictive macrostate trajectories to obtain a Gaussian posterior distribution for macrostates.
(4) Sample a new ensemble of macrostates from the posterior distribution obtained from the EnKF.
(5) For each member of the new macrostate ensemble synthesize a compatible microstate.

A diagram of this procedure is shown in figure 1a. Step 5 requires additional explanation. How do we generate these microstates? Both intuition and our physics analogy suggest that whatever microstate μ we choose to represent the macrostate M should be one which is realistic in the sense that it is highly likely for the model to be in that state. That is, we would like to choose μ based on the probability distribution p(μ|M) . For simple models, it may be possible to describe all or parts of p(μ|M) explicitly, e.g. agent positions and velocities in the WSG model. In that case, we can generate μ by sampling from said distribution. However, as the complexity of the model increases, our ability to describe p(μ|M) explicitly is reduced and we must develop other methods.

The methods for microstate synthesis that we present here are based on the following principles and assumptions:

— Continuity: If $M_{0}$ and $M_{1}$ are nearby macrostates, then the distributions $p(\mu |M_{0})$ and $p(\mu |M_{1})$ are similar. That is, if $\mu_{0}$ is a microstate for $M_{0}$ , then there is a microstate $\mu_{1}$ for $M_{1}$ with a similar likelihood and which is close to $\mu_{0}$ (see figure 1b).
— Local similarity: In spatial models, agents in similar local neighbourhoods should have similar trajectories.

The continuity assumption suggests the following algorithm. Given a collection $\left\{{\hat{\mu }}_{i}\right\}$ of predictive microstates and $\{{\hat{M}}_{i}{\}}_{i}$ the corresponding collection of macrostates and $\left\{M_{i}\right\}$ a sample from the KF posterior, pick a pairing ${\hat{M}}_{i}↔M_{i}$ , which minimizes pairwise change. (We use the Gale–Shapley algorithm.) The continuity assumption then tells us that there should be microstates $\left\{\mu_{i}\right\}$ for the macrostates $\left\{M_{i}\right\}$ that are pairwise close to the $\left\{{\hat{\mu }}_{i}\right\}$ . Thus, the problem becomes: how do we synthesize a microstate $\mu_{i}$ for $M_{i}$ , given a similar microstate ${\hat{\mu }}_{i}$ which has a similar macrostate ${\hat{M}}_{i}$ ?

By passing to the macrostate, we resolve the first and second problems associated with applying KF methods to ABMs. Recall the third of our problems: that an agent’s state may not be a vector space. We propose a generalizable solution for agents taking a categorical state and implement it, and a corresponding microstate synthesis algorithm which respects non-uniform spatial distributions, for epithelial cells in the viral dynamics model. This approach can be applied to models in which agents positioned on a grid can take one of several categorical states.

For the fourth problem, the dimensionality of spatial components, we note that naively viewing a density field as a vector exploits none of the spatial relations of the model such as similarity between local neighbourhoods or the possible spatial symmetries of the model. Both the WSG and viral dynamics models are invariant under affine transformations of the plane. For this reason, we enhance our quantization error-diffusion algorithm to also impute molecular field levels based on local data. For details, see the section on Quantization and Error Diffusion in the electronic supplementary material.supplementary material.

### 3.3. Spatially well-mixed predator–prey dynamics

Our first example is a relatively simple ABM of predator–prey dynamics, the Wolf-Sheep predation model [51,78,79], a member of the Netlogo [80] model library which we have re-implemented in Python [81] under the name of the WSG model. In this model, wolves, sheep and grass exist in a two-dimensional space. Grass occupies fixed patches in this space while wolves and sheep move through space in a random walk. Each grass pixel can be either on or off, indicating the presence of grass and wolves and sheep keep an internal energy counter. Wolves, resp. sheep, eat sheep, resp. grass, at their location, each gaining energy in the process, and grass regrows over time. Wolves and sheep both reproduce randomly, with some probability, splitting their energy between the parent and child (see table 1 for a summary of the variables present in the model).

**Table 1.** Micro- and macrostate variables for the WSG model. Each wolf and sheep agent has a position, velocity and energy while grass agents are described by their growth state (green/brown) and regrowth timer. Macrostate summaries of these agents are given by the count of wolf and sheep agents and the count of grass agents in the alive state.

variables		microstate	macrostate
per-agent	wolf	position	count
		velocity	
		energy	
	sheep	position	count
		velocity	
		energy	
spatial	grass	growth state	count
		regrowth counter	

As seen in table 1, we choose to describe a macrostate of this model in terms of the number of wolves, sheep and proportion of grass coverage. As both wolves and sheep move in a random walk, their observed spatial distributions are approximately uniform, as is the distribution of grass. This suggests that once a macrostate has been specified, it is straightforward to construct a likely spatial distribution of wolves, sheep and grass by uniform random sampling.

As described above, KF methods require some degree of tuning to achieve good performance. With the components of the macrostate chosen, the next type of tuning to consider consists of variable transformations. In the WSG model, it is relatively common for wolves to go extinct and so the support for the wolf distribution is often concentrated around zero. For this reason, we make a variable transformation on the number of wolves, $W^{\prime }=\log\;W$ . Note that this itself adds a certain degree of complication, since the number of wolves can actually be zero. We resolve this by using a slight variant of the log⁡ transform, instead working with $W^{\prime }=\log(\epsilon +W)$ and the inverse transform $W=\max(0.0,\exp(W^{\prime })-\epsilon )$ where ϵ=0.001 *.*

As the KF is a minimum least squares estimator, the KF tends to learn the largest magnitude quantities very strongly while treating other interesting, but lower magnitude, parameters more like noise. This means that variable transformations, such as scaling, may also be required to achieve good performance of the KF. Notably, the number of sheep and grass are typically one and two orders of magnitude more than the number (or log number) of wolves, respectively, while the parameters tend to be around the same order of magnitude as the log⁡ -wolves. For this reason, we scale the number of sheep by 0.1 and the grass count by 0.01 to keep these variables in similar ranges.

**Figure 2.**
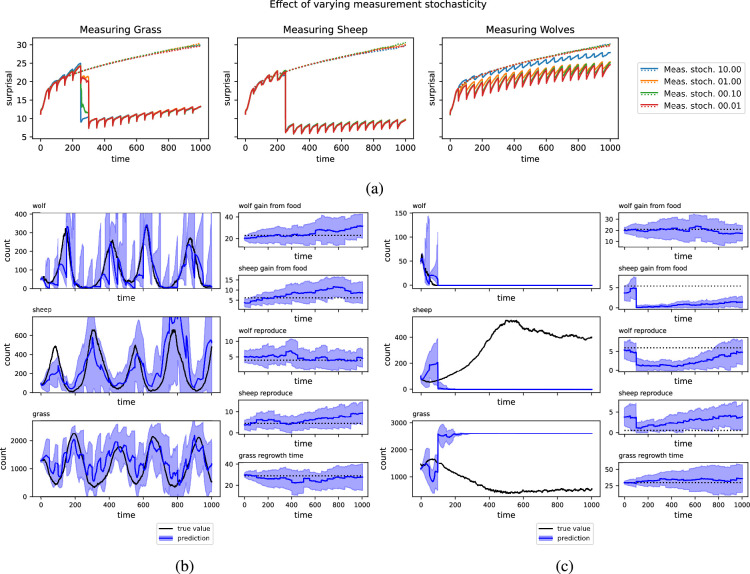
(a) Median surprisal over 1000 runs as a time series for the original ensemble and after a series of KF update steps. Measurements taken on wolves, sheep and grass, respectively. Downward spikes in surprisal correspond to KF updates. Q=0.01 . Spatial dimensions = 255×255 . (b) A typical run of the ABMKF algorithm where measurements are taken on the count of wolves. In black, the true trajectory. In blue, the distribution of trajectories learned, with the uncertainty cone. The corrections to the wolf count are close to the true value with a corresponding narrowing of the uncertainty bands after measurement. The amount of narrowing and degree to which the mean moves to the observation depend on the measurement uncertainty. The KF similarly corrects the sheep and grass counts reasonably well, despite these counts not being directly measured. As such, the uncertainty bands around these predictions do not change as much. (c) A very high surprisal trajectory. In black, the true trajectory. In blue, the distribution of trajectories learned, with uncertainty cone. In the true trajectory, wolves have gone extinct and are the sole measurement being taken. In this system, there are two possible equilibria consistent with wolf extinction: one where the sheep stabilize around a high level, and another where the sheep are also extinct. In this case, the KF stabilized around the extinct-sheep fixed point, but the system is near the high-sheep attractor. This incorrect choice cannot be corrected by further wolf measurements.

Measurement uncertainty was tuned by simulation with the following procedure. For each choice of measurement and measurement uncertainty, we generate a population of virtual trajectories from the model. That is, we generate a sample of random parametrizations of the WSG model and run each through 1000 updates to form a population of true trajectories. We then run the KF methods described above, with the chosen measurement taken at regular intervals, and evaluate the data-assimilation process using surprisal. With 1000 sample simulations each, we evaluated the three basic measurements (i.e. wolves, sheep or grass) and four levels of measurement uncertainty (i.e. 10.0 , 1.0 , 0.1 and 0.01 ). The results are shown in figure 2a. Note that the lowest level tested generally, but not always, performed best.

Process stochasticity was also tuned by simulation in a similar manner. Note that here we only add process stochasticity to the parameters, as the computational model includes inherent stochasticity for the state variables. Also, the added stochasticity is only added to the model instances used in the KF; the parameters of the true trajectory are kept constant (see electronic supplementary material, figure S1).

In figure 2b,c, we show examples of trajectories learned. In figure 2b, measurements are taken on the number of sheep and we see a relatively good example of the KF correcting errors in prediction. While the KF incorrectly predicts that wolves will go extinct, leading to upward wandering predictions on the number of sheep, measurements on the number of sheep keep the overall prediction from completely diverging from the true trajectory. However, figure 2c shows a failure mode for KF methods. In this example, the KF is attempting to learn from periodic measurements of wolves, but in fact wolves have gone extinct. While the KF successfully learns that the number of wolves is zero, it strongly misidentifies the state of sheep and grass. That is, the wolf-free model has two attractor states: one which is a random walk around a large number of sheep and small quantity of grass and another in which sheep are extinct and grass is at a steady state. In figure 2c, the random chance of sampling yields the incorrect option and, due to the unimodal nature of Gaussian distributions, gets stuck there. This example serves to demonstrate the importance of measurement type as either additional sheep or grass measurements could distinguish between these attractors.

### 3.4. Spatially heterogeneous viral dynamics

The second ABM, which we will examine is a model of human anti-viral immune responses [52] whose basic structure is relevant to the ICU patient described earlier. This model is significantly more complex than the WSG model, both in the number of agents and parameters and in the behaviour of the agents. In this model, spatial distributions of agents are distinctly non-uniform and play a significant role in the model’s evolution.

Briefly, the model is spatial, on a two-dimensional lattice of size 51×51 . It contains several immune cells as mobile agents: dendritic cells, pulmonary neutrophils, macrophages and natural killer cells. Fixed at each lattice point, the model includes agents modelling the epithelium and endothelium and the quantity of extracellular virus and various molecular species. The human portion of the model has approximately 60 parameters and 42 microstate variables, which partially correspond to 20 macrostate variables; see electronic supplementary material, table S1. A sensitivity analysis selects 19 of the 60 parameters; see the electronic supplementary material for more detail.

The dimension of a microstate for this model is sizable from the perspective of DA, with 20 variables at each of the 51×51 lattice sites. Each of the mobile agents contributes at least a location (two-dimensional) and movement direction (two-dimensional) to the microstate as well as other fields depending on the type of agent. In our Python implementation of this model, on a typical 64-bit computer, the microstate takes around 0.95 megabytes of memory, depending somewhat on the number of agents. This puts us in the regime where it is possible to hold many instances of the model in memory simultaneously, but where inference from low-dimensional data is challenging.

Among the possible macrostate components, we consider several which could potentially be measurable using non-destructive procedures such as a bronchoalveolar lavage or through blood work. In particular, we select 22 quantities to describe the macrostate of the model: total quantities of all modelled molecules, amounts of extracellular and intracellular virus, counts of lung epithelial and endothelial cells in various states, counts of the various immune cell agents and a count of the number of apoptotic epithelial cells which have been killed by the virus. For this introductory study, we measure these values directly from the simulation.

In the WSG model, the distributions of wolves, sheep and grass were close to uniform and we were able to reconstruct compatible microstates by simple random sampling. However, in the viral dynamics model, spatial distributions of cytokines and cells are highly non-uniform and are characterized by ‘hot-spots’, where the infection is present. This complicates the synthesis of compatible microstates. We developed and tested two microstate synthesis algorithms. The first is a simple update technique similar to that which was used for the WSG model. The second is a more complex, spatially aware technique that makes modifications that try to maximize the similarity of local neighbourhoods to previously seen local neighbourhoods.

For some model quantities, there are simple methods to update microstates. For example, in the case of a molecular field, such as IL1, an update to the total_IL1 macrostate can be propagated to the IL1 microstate by appropriate scaling. This method preserves spatial correlations. However, this method does not work for all model quantities. For example, consider the update of the counts of epithelial cells. In this model, epithelial cells are located at patches and every patch in the simulation can take several categorical states in which the patch is empty or contains a healthy, an infected, or dead epithelial cell. Dead epithelial cells are distinguished by process: apoptosis or necrosis.

The macrostate update may change the total number of cells in each category but the total number in all categories remains constant and equal to the number of patches. In the simpler of the microstate synthesis algorithms, we change the microstate to the desired macrostate by altering the states of randomly selected cells. This achieves a certain type of minimality to the microstate update: it minimizes the total number of changed epithelial states.

Since this random alteration is not chosen with regard to the geometry of the infection, we have no expectation that this manner of update will preserve the hot spots. In fact, we typically see that this form of update will tend to create new hot spots as newly placed infected epithelial cells will often be outside of existing infected regions (see figure 1b).

To remedy this problem, we introduced an update technique which better preserves the existing geometry of a microstate. Consider the case of the categorical state of epithelial cells in the viral dynamics model. The existing technique of scaling, which we performed for molecular concentrations, cannot be done directly on a categorical state. Instead we developed a three-step process: (i) encode each cell’s categorical state as a vector, through what is known as one-hot encoding in machine learning [74] or as a dummy-variable in statistics, (ii) scale the resulting vector field to be compatible with the desired macrostate, and (iii) quantize the vector field back to a categorical state while preserving the global counts of the various categories. In more detail:

(1) *One-hot encoding of the previous state.* Each patch’s state takes values in one out of five categories: empty, or containing a healthy, infected, necrotic or apoptosed epithelial cell. The one-hot encoding is then a mapping of these categorical states to the standard basis vectors of ${\mathbb{R}}^{5}$ : Healthy $\to e_{1}$ , Infected $\to e_{2}$ , Necrosed $\to e_{3}$ , Apoptosed $\to e_{4}$ , Empty $\to e_{5}$ . Note that the counts of each type are now the components of the vector obtained by summing the one-hot encoded vectors (see figure 1c).
(2) *Rescaling.* Given the initial epithelial-state count macrostate (H,I,D,A,E) and updated epithelial-state count macrostate $\left(H^{\prime },I^{\prime },D^{\prime },A^{\prime },E^{\prime }\right)$ *,* we rescale the one-hot vectors by component-wise multiplication by $\left(H^{\prime }/H,I^{\prime }/I,D^{\prime }/D,A^{\prime }/A,E^{\prime }/E\right)$ . These new vectors are no longer one-hot encoded, but do have the property that the sum of sites will have the updated macrostate $\left(H^{\prime },I^{\prime },D^{\prime },A^{\prime },E^{\prime }\right)$ (see figure 1c).
(3) *Quantization and error diffusion.* The goal of this step is to return the epithelial vectors to a one-hot encoding while preserving the updated macrostate. Our procedure for this involves two steps. First, based on the state vector $\vec{s}$ of each epithelial cell and possibly its surroundings, we must choose an appropriate *quantization* to one of the standard basis vectors $e_{i}$ . These algorithms preserve various qualitative and quantitative properties and have an almost 100-year-long history in image processing [82,83]. Instead of optimizing quantization for visual perception, we will base the quantization on the minimum of a multi-component ‘loss function’ which balances various targets:

$$L(q)=\lambda_{1}L_{1}(q)+\lambda_{2}L_{2}(q)+\lambda_{3}L_{3}(q),$$

where $L_{i}(q)$ are target functions related to quantization error, neighbourhood structure, and neighbourhood likelihood, respectively, and $\lambda_{i}$ are scalar weights. See the section on Quantization and Error Diffusion in the electronic supplementary material for an explicit definition of each target function. This quantization introduces an error term, $\Delta =q-\vec{s}$ , to the macrostate. This error is then uniformly *diffused* to any surrounding unquantized epithelial cells (see electronic supplementary material, figure S2). By distributing the error to surrounding epithelial cells, we preserve the overall macrostate.

We repeat this three-step process for the endothelial cells as well, which have three categorical states: normal, activated and dead.

As stated above, there are multiple options for how to appropriately choose a quantization and multiple options for the pattern of error diffusion, each of which can be arbitrarily paired in the overall quantization/error-diffusion algorithm. We consider here an alternative to choosing the closest quantization, which incorporates data from the local neighbourhood. We seek a quantization that seeks to minimize a weighted combination of three things:

(1) The quantization error, represented by ${\left|q-\vec{s}\right|}^{2}$ . This influences the quantization to be close to $\vec{s}$ .
(2) The dissimilarity of the quantization to neighbours, represented by the negative logarithm of c⋅q , where c is a regularized count of the number of each type of cell in a 3×3 neighbourhood. This influences the quantization to be similar to neighbouring values, reducing the number of isolated types.
(3) The negative log⁡ probability of the neighbourhood based on previous runs. This measure, which is based on both local cells states and local cytokine levels, influences the algorithm to pick quantizations that have been previously observed.

See the electronic supplementary material for further details.

**Figure 3.**
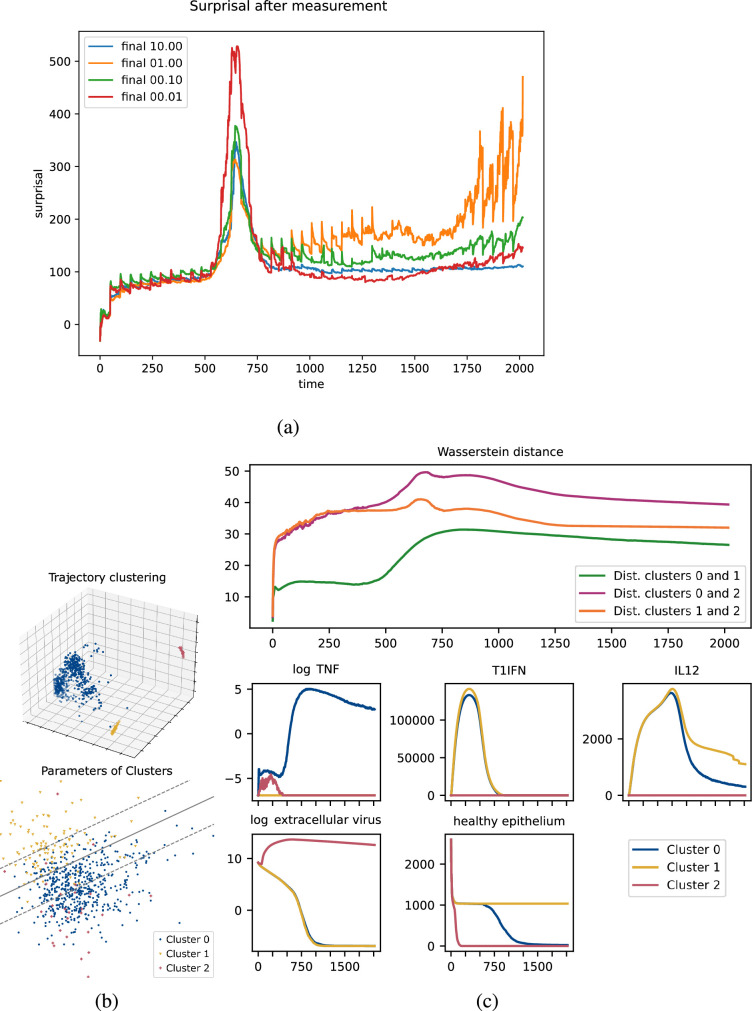
(a) Tuning the measurement uncertainty for the viral dynamics model: median surprisal over 695 simulations (random parametrizations) as a time series of 2016 updates incorporating the KF update. In the spike from t=500 to t=750 , the curve with the lowest measurement uncertainty has the highest surprisal due to overfitting by the ABMKF during a period of divergence in trajectories. (b) Dimensional reduction of trajectories using PCA shows clusters that suggest phenotypes. Top: trajectories, suggesting three clusters, corresponding to possible phenotypes. Bottom: parameters, labelled by cluster, showing approximate linear boundary between clusters 0 and 1 . Separating lines computed using scikit-learn's LinearSVC. (c) Top plot: time series of Wasserstein distances between clusters. At each time-point, the Gaussian approximation of each cluster is calculated and the pairwise Wasserstein distances are calculated between the resulting three Gaussians. Cluster 2 distinguishes itself rapidly from the others, while clusters 0 and 1 have an abrupt separation between t=500 and t=750 . Lower rows show a representative selection of macrostate components. TNF (shown in log⁡ levels), P/DAMPS, IL1, IL6 and IL10 are present at low levels in both clusters 0 and 1 until the clusters diverge around t=500 . Similarly, healthy epithelial counts also distinguish these clusters at around this time. Clusters 0 and 1 have similar trajectories in T1IFN (shown) and IFNg for the entire course of the infection, while these clusters have divergent IL12 (shown) and IL8 levels in the later stage of the infection. Cluster 2 is distinguished by extremely high levels of virus (shown in log⁡ levels), and rapid epithelial cell death.

Initial tuning of the KF for the viral dynamics model revealed interesting effects across 695 simulations; each simulation had a random parametrization and ran for 2016 updates. Notably, there is a spike in uncertainty from around t=500 to t=750 when tuning the measurement uncertainty and a similar but longer lasting spike when tuning the process stochasticity. In each case, the spike in uncertainty is resolved after further time and measurements. See figure 3a for measurement stochasticity and electronic supplementary material, figure S3, for process stochasticity.

To explore what led to this phenomenon, we collated the virtual patient trajectories generated while running the tuning process. This resulted in 695 simulation runs consisting of 41 variables sampled at 2017 time points. These were flattened into 695 samples in a 2017×41 dimensional space and principal component analysis (PCA) was used to dimensionally reduce the vectors into 3 dimensions. See the left subfigure in figure 3b. This reduction showed that the trajectories fall into at least 3 meaningfully distinct clusters, suggesting a possible decomposition of the trajectories into phenotypes. To evaluate the possibility that these conjectured phenotypes are driven by model parametrization, we performed PCA on our 695 samples to reduce the 19-dimensional model parametrizations to 3 dimensions. The first dimension of this PCA was found to be an uninformative artefact of rounding certain parameters to integer values. The second and third dimensions are used to plot the left subfigure in figure 3b. This 2-dimensional plot shows a rough, but distinct, linear separation pattern between clusters 0 and 1 which corresponds to a separating hyperplane in the full 19-dimensional parameter space. This confirms that these clusters are in part driven by model parametrizations. Note that cluster 2, having only 28 representatives, does not have a clear pattern with respect to this hyperplane.

In figure 3c, we see some justification for the idea that these clusters drive the spike in surprisal and correspond to a divergence between phenotypes at that time. Consider the top subfigure in figure 3c in which we see the time-wise change between each cluster in terms of Wasserstein distance [84], a well-established measure of the difference between probability distributions. Cluster 2 separates quickly from the others, which can be seen from the rapid growth and sustained height of the orange and purple curves. The distance between clusters 0 and 1, however, remains comparatively low until around t=500 , transitioning to a sustained higher level by t=750 . This change should be interpreted as a divergence in the phenotypes in that region. In effect, we see three phases for the surprisal. First, a lower surprisal phase where the phenotypes are not very distinct. Second, a higher surprisal phase, where the phenotypes diverge and the KF is uncertain which corresponds to the virtual patient. Finally, a third phase, which returns to lower surprisal levels as the KF has resolved the virtual patient’s phenotype. This interpretation is backed up by examining the mean cluster trajectories, as seen in the lower plots of figure 3.

This clustering phenomenon, corresponding to the divergence of phenotypes, brings up an interesting point. As noted in [85], the KF is an optimal minimum mean square error estimator (MMSEE), regardless of the underlying distribution type. In that article, the main thrust is that the optimality of the KF does not depend on the Gaussian assumption and that the KF is then an optimal choice in more situations than commonly believed. However, the diverging phenotypes seen above are exactly the sort of phenomenon in which an MMSEE is not a reasonable estimate. This type of estimator is most appropriate for unimodal data; when there are multiple clusters, an MMSEE will often predict something in the low-likelihood region in-between the clusters.

## 4. Discussion

In this work, we introduced a microstate synthesis algorithm that allows us to apply KF methods to ABMs. As a first application, we applied this algorithm to a predator–prey model. Given that the model was spatially well mixed, we chose a simple approach for microstate synthesis in which agent generation and removal actions were carried out using uniform random distributions. With this approach, our algorithm performed well, with notable exceptions occurring when virtual patients and/or ensemble members ran into boundary states where one or more agent types went extinct. We then applied our DA algorithm to a viral dynamics model. Because the spatial heterogeneity of this model strongly influences system dynamics, we opted for a more spatially aware approach to microstate synthesis. This spatially aware microstate synthesis algorithm, which carries out the continuous Kalman update in discrete settings using a quantization algorithm, performed well overall. At the same time, the discovery of a spike in surprisal revealed how the KF performs when the model contains several phenotypes which diverge mid-simulation.

An important direction for our future work is to study the general applicability of this personalization method and required modifications or extensions beyond the two use cases we presented here. One major challenge is that there is great diversity in the structure, features and implementations of ABMs, and an absence of standards for their specification and implementation. Another challenge involves modelling a measurement procedure itself, such as the location of blood draw and analytic device used [86–88]. As the two use cases show, even for relatively simple models, application and analysis of our algorithms is complex. In part, we will address this issue by focusing on models implemented in one of the standard modelling platforms, including CompuCell3D [21], TissueForge [23], PhysiCell [22] and others. This introduces a certain standardization of model structure and opens the possibility of standardizing at least part of our algorithm.

In other future work, we will introduce a technique to deal with the kind of no-exit sets that were observed in the WSG model. This extension, which we have given the preliminary name of ‘stratified Kalman filter’, partitions the state space into strata based on the no-exit sets, keeping track of the likelihood that the model is in each stratum while performing KF techniques within each stratum. We have also begun work on improving microstate synthesis using modern AI techniques such as generative adversarial networks and diffusion models. These generative models allow us to view microstate synthesis as a sampling procedure from the distribution of microstates conditional on a macrostate, rather than requiring an existing realistic microstate to update. The ability to generate realistic microstates directly from a macrostate will allow several improvements, including the ability to start the patient at an arbitrary time without requiring a specific initializing configuration. Additionally, we are developing techniques to better deal with phenotype-based nonlinearities in systems such as those seen in the viral dynamics model. The detection of these phenotypes provides valuable qualitative information for a physician but requires additional technique development beyond of already established technology, including the ability to account for multi-modal, per-phenotype distributions.

We expect that, when further expanding the applications of the ABMKF to stochastic *multiscale* models, such as a model [14,89] our laboratory developed to study the immune response to respiratory fungal infections, all of the above issues will persist. Such models will require further new techniques which can bridge the various scales. Another issue which we did not fully explore in this work involves the question of whether it is necessary for microstate synthesis to preserve the spatial correlations present in microstates to produce likely microstate updates and improve model predictions. It has been observed that spatial correlations in ABM microstates can and do influence future model outcomes; see [90] for example, which includes explicit measurements of such correlations. In our work, preliminary results incorporating such correlations into the loss function of our microstate synthesis algorithm (specifically in the quantization and error-diffusion step) did not improve model predictions for the viral dynamics model. Nonetheless, it may be the case that other ABMs may be more sensitive to such spatial correlations, meaning that preserving these correlations may be necessary for synthesizing microstates to yield accurate model predictions. We are incorporating this question into the AI-based microstate synthesis techniques which we are developing.

As we have discussed above, the DA tools developed in this article could serve as one component of a digital twin. Such a twin can serve as a virtual experimental setup where a physician can test possible medical interventions against the predicted effects on the personalized digital twin. This human-in-the-loop setup can be expanded to consider actions other than strictly therapeutic treatments, including the question of which measurements to take. That is, the model could provide guidance as to which measurements will be most informative at any given time, allowing the digital twin to advise physicians how proposed measurements will affect predictions and either order or skip certain diagnostics. Tools have been developed in control theory [91,92] to answer exactly these measurement questions, but mostly targeted to the case where there is no human in the loop and the decision of what to measure is answered once and for all. In future work, we will explore the human-in-the-loop aspect of measurement and the question of how best to offer guidance to the user on all of the available actions.

## Data Availability

Implementation details, code and data are freely available on GitHub under the MIT licence. The state of the code at publishing has been permanently archived at [93] and an active git repository is at [94]. Electronic supplementary material is available online [95].

## References

[1] Elayan H , Aloqaily M , Guizani M . 2021 Digital twin for intelligent context-aware IoT healthcare systems. IEEE Internet Things J. **8** , 16749–16757. (10.1109/jiot.2021.3051158)

[2] Alazab M *et al* . 2023 Digital twins for healthcare 4.0—recent advances, architecture, and open challenges. IEEE Consum. Electron. Mag. **12** , 29–37. (10.1109/mce.2022.3208986)

[3] Okegbile SD , Cai J , Niyato D , Yi C . 2023 Human digital twin for personalized healthcare: vision, architecture and future directions. IEEE Netw. **37** , 262–269. (10.1109/mnet.118.2200071)

[4] Okegbile SD , Cai J , Wu J , Chen J , Yi C . 2024 A prediction-enhanced physical-to-virtual twin connectivity framework for human digital twin. IEEE Trans. Cogn. Commun. Netw. 1–1. (10.1109/tccn.2024.3519331)

[5] Breton MD *et al* . 2020 A randomized trial of closed-loop control in children with type 1 diabetes. N. Engl. J. Med. **383** , 836–845. (10.1056/nejmoa2004736) 32846062 PMC7920146

[6] National Academy of Engineering and National Academies of Sciences, Engineering, and Medicine . 2023 Opportunities and challenges for digital twins in biomedical research (ed. L Casola ). In Proc. of a Workshop–in Brief. Washington, DC: The National Academies Press. https://nap.nationalacademies.org/catalog/26922/opportunities-and-challenges-for-digital-twins-in-biomedical-research-proceedings. 37339242

[7] National Academy of Engineering and National Academies of Sciences, Engineering, and Medicine . 2024 Foundational research gaps and future directions for digital twins. Washington, DC: The National Academies Press. See https://nap.nationalacademies.org/catalog/26894/foundational-research-gaps-and-future-directions-for-digital-twins. 39088664

[8] Masison J *et al* . 2021 A modular computational framework for medical digital twins. Proc. Natl Acad. Sci. USA **118** , e2024287118. (10.1073/pnas.2024287118) 33972437 PMC8157963

[9] Wieland FG , Hauber AL , Rosenblatt M , Tönsing C , Timmer J . 2021 On structural and practical identifiability. Curr. Opin. Syst. Biol. **25** , 60–69. (10.1016/j.coisb.2021.03.005)

[10] Glen CM , Kemp ML , Voit EO . 2019 Agent-based modeling of morphogenetic systems: advantages and challenges. PLoS Comput. Biol. **15** , e1006577. (10.1371/journal.pcbi.1006577) 30921323 PMC6438454

[11] Buttenschön A , Edelstein-Keshet L . 2020 Bridging from single to collective cell migration: a review of models and links to experiments. PLoS Comput. Biol **16** , e1008411. (10.1371/journal.pcbi.1008411) 33301528 PMC7728230

[12] Montagud A , Ponce-de-Leon M , Valencia A . 2021 Systems biology at the giga-scale: large multiscale models of complex, heterogeneous multicellular systems. Curr. Opin. Syst. Biol. **28** , 100385. (10.1016/j.coisb.2021.100385)

[13] Pleyer J , Fleck C . 2023 Agent-based models in cellular systems. Front. Phys **10** , 968409. (10.3389/fphy.2022.968409)

[14] Adhikari B *et al* . 2022 Computational modeling of macrophage iron sequestration during host defense against Aspergillus. mSphere **7** , e0007422. (10.1128/msphere.00074-22) 35862797 PMC9429928

[15] Segovia-Juarez JL , Ganguli S , Kirschner D . 2004 Identifying control mechanisms of granuloma formation during M. tuberculosis infection using an agent-based model. J. Theor. Biol. **231** , 357–376. (10.1016/j.jtbi.2004.06.031) 15501468

[16] Jenner AL *et al* . 2022 Agent-based computational modeling of glioblastoma predicts that stromal density is central to oncolytic virus efficacy. iScience **25** , 104395. (10.1016/j.isci.2022.104395) 35637733 PMC9142563

[17] Fertig EJ , Jaffee EM , Macklin P , Stearns V , Wang C . 2021 Forecasting cancer: from precision to predictive medicine. Med **2** , 1004–1010. (10.1016/j.medj.2021.08.007) 35330848 PMC8942083

[18] West J , Robertson-Tessi M , Anderson ARA . 2023 Agent-based methods facilitate integrative science in cancer. Trends Cell Biol. **33** , 300–311. (10.1016/j.tcb.2022.10.006) 36404257 PMC10918696

[19] Metzcar J , Wang Y , Heiland R , Macklin P . 2019 A review of cell-based computational modeling in cancer biology. JCO Clin. Cancer Inform. **3** , 1–13. (10.1200/CCI.18.00069) PMC6584763 30715927

[20] Cogno N , Axenie C , Bauer R , Vavourakis V . 2024 Agent-based modeling in cancer biomedicine: applications and tools for calibration and validation. Cancer Biol. Ther. **25** , 2344600. (10.1080/15384047.2024.2344600) 38678381 PMC11057625

[21] Swat MH , Thomas GL , Belmonte JM , Shirinifard A , Hmeljak D , Glazier JA . 2012 Multi-scale modeling of tissues using CompuCell3D. In Methods in cell biology computational methods in cell biology (eds AR Asthagiri , AP Arkin ), pp. 325–366. Academic Press. (10.1016/b978-0-12-388403-9.00013-8) PMC3612985 22482955

[22] Ghaffarizadeh A , Heiland R , Friedman SH , Mumenthaler SM , Macklin P . 2018 PhysiCell: an open source physics-based cell simulator for 3-D multicellular systems. PLoS Comput. Biol. **14** , e1005991. (10.1371/journal.pcbi.1005991) 29474446 PMC5841829

[23] Sego TJ , Sluka JP , Sauro HM , Glazier JA . 2023 Tissue forge: interactive biological and biophysics simulation environment. PLoS Comput. Biol. **19** , e1010768. (10.1371/journal.pcbi.1010768) 37871133 PMC10621971

[24] Starruß J , de Back W , Brusch L , Deutsch A . 2014 Morpheus: a user-friendly modeling environment for multiscale and multicellular systems biology. Bioinformatics **30** , 1331–1332. (10.1093/bioinformatics/btt772) 24443380 PMC3998129

[25] Kovatchev BP , Breton M , Dalla Man C , Cobelli C . 2009 In silico preclinical trials: a proof of concept in closed-loop control of type 1 diabetes. J. Diabetes Sci. Technol. **3** , 44–55. (10.1177/193229680900300106) 19444330 PMC2681269

[26] Jenner AL *et al* . 2021 COVID-19 virtual patient cohort suggests immune mechanisms driving disease outcomes. PLoS Pathog **17** , e1009753. (10.1371/journal.ppat.1009753) 34260666 PMC8312984

[27] Tang D , Malleson N . 2022 Data assimilation with agent-based models using Markov chain sampling. Open Res. Eur. **2** , 70. (10.12688/openreseurope.14800.1)

[28] Monti C , Pangallo M , De Francisci Morales G , Bonchi F . 2023 On learning agent-based models from data. Sci. Rep. **13** , 9268. (10.1038/s41598-023-35536-3) 37286576 PMC10247821

[29] Ngom B , Diallo M , Marilleau N . 2020 MEDART-MAS:MEta-model of data assimilation on real-time multi-agent simulation. In Int. Symp. on Distributed Simulation and Real Time Applications, Prague, Czech Republic, 14-16 September 2020. (10.1109/DS-RT50469.2020.9213694)

[30] Ternes P , Ward JA , Heppenstall A , Kumar V , Kieu LM , Malleson N . 2021 Data assimilation and agent-based modelling: towards the incorporation of categorical agent parameters. Open Res. Eur. **1** , 131. (10.12688/openreseurope.14144.2) 37645182 PMC10445938

[31] Suchak K , Kieu M , Oswald Y , Ward JA , Malleson N . 2024 Coupling an agent-based model and an ensemble Kalman filter for real-time crowd modelling. R. Soc. Open Sci. **11** , 231553. (10.1098/rsos.231553) 38623082 PMC11017988

[32] Malleson N , Minors K , Kieu LM , Ward JA , West A , Heppenstall A . 2020 Simulating crowds in real time with agent-based modelling and a particle filter. J. Artif. Soc. Soc. Simul. **23** , 3. (10.18564/jasss.4266)

[33] Kelly C , Michelsen FA , Alver MO . 2023 An ensemble modelling approach for spatiotemporally explicit estimation of fish distributions using data assimilation. Fish. Res. **261** , 106624. (10.1016/j.fishres.2023.106624)

[34] Clay R , Ward JA , Ternes P , Kieu LM , Malleson N . 2021 Real-time agent-based crowd simulation with the reversible jump unscented Kalman filter. Simul. Model. Pract. Theory **113** , 102386. (10.1016/j.simpat.2021.102386)

[35] Clay R , Kieu LM , Ward JA , Heppenstall A , Malleson N . 2020 Towards real-time crowd simulation under uncertainty using an agent-based model and an unscented Kalman filter. In Advances in practical applications of agents, multi-agent systems, and trustworthiness. The PAAMS collection (eds Y Demazeau , T Holvoet , JM Corchado ), pp. 68–79. Cham, Switzerland: Springer International Publishing. (10.1007/978-3-030-49778-1_6)

[36] Kieu LM , Malleson N , Heppenstall A . 2020 Dealing with uncertainty in agent-based models for short-term predictions. R. Soc. Open Sci. **7** , 191074. (10.1098/rsos.191074) 32218939 PMC7029931

[37] Ward JA , Evans AJ , Malleson NS . 2016 Dynamic calibration of agent-based models using data assimilation. R. Soc. Open Sci. **3** , 150703. (10.1098/rsos.150703) 27152214 PMC4852637

[38] Makinoshima F , Oishi Y . 2022 Crowd flow forecasting via agent-based simulations with sequential latent parameter estimation from aggregate observation. Sci. Rep. **12** , 11168. (10.1038/s41598-022-14646-4) 35778445 PMC9249888

[39] Ghorbani A , Ghorbani V , Nazari-Heris M , Asadi S . 2023 Data assimilation for agent-based models. Mathematics **11** , 4296. (10.3390/math11204296)

[40] Wang M , Hu X . 2013 Data assimilation in agent based simulation of smart environment. In Conf. on Principles of Advanced Discrete Simulation, *New York, NY, USA* , vol. 2013, pp. 379–384, (10.1145/2486092.2486145)

[41] Robin TT , Cascante-Vega J , Shaman J , Pei S . 2024 System identifiability in a time-evolving agent-based model. PLoS ONE **19** , e0290821. (10.1371/journal.pone.0290821) 38271401 PMC10810497

[42] Tabataba FS , Lewis B , Hosseinipour M , Tabataba FS , Venkatramanan S , Chen J , Higdon D , Marathe M . 2017 Epidemic forecasting framework combining agent-based models and smart beam particle filtering. In 2017 IEEE Int. Conf. on Data Mining (ICDM), *New Orleans, LA, USA* , pp. 1099–1104. (10.1109/ICDM.2017.145)

[43] Sun C , Richard S , Miyoshi T , Tsuzu N . 2022 Analysis of COVID-19 spread in Tokyo through an agent-based model with data assimilation. J. Clin. Med. **11** , 2077–0383. (10.3390/jcm11092401) 35566527 PMC9103055

[44] Asher M , Lomax N , Morrissey K , Spooner F , Malleson N . 2023 Dynamic calibration with approximate Bayesian computation for a microsimulation of disease spread. Sci. Rep. **13** , 8637. (10.1038/s41598-023-35580-z) 37244962 PMC10221755

[45] Rai S , Hu X . 2018 Building occupancy simulation and data assimilation using a graph-based agent-oriented model. Physica A **502** , 270–287. (10.1016/j.physa.2018.02.051)

[46] Cocucci TJ , Pulido M , Aparicio JP , Ruíz J , Simoy MI , Rosa S . 2022 Inference in epidemiological agent-based models using ensemble-based data assimilation. PLoS One **17** , e0264892. (10.1371/journal.pone.0264892) 35245337 PMC8896713

[47] Schneider T *et al* . 2022 Epidemic management and control through risk-dependent individual contact interventions. PLoS Comput. Biol **18** , e1010171. (10.1371/journal.pcbi.1010171) 35737648 PMC9223336

[48] Fonseca LL , Böttcher L , Mehrad B , Laubenbacher RC . 2025 Optimal control of agent-based models via surrogate modeling. PLoS Comput. Biol **21** , e1012138. (10.1371/journal.pcbi.1012138) 39808665 PMC11790234

[49] Böttcher L , Fonseca LL , Laubenbacher RC . 2025 Control of medical digital twins with artificial neural networks. Phil. Trans. R. Soc. A **383** , 20240228. (10.1098/rsta.2024.0228) 40078154 PMC11904622

[50] Evensen G . 1994 Sequential data assimilation with a nonlinear quasi‐geostrophic model using Monte Carlo methods to forecast error statistics. J. Geophys. Res. **99** , 10143–10162. (10.1029/94jc00572)

[51] Wilensky U . 1997 NetLogo Wolf Sheep Predation model. Northwestern University, Evanston, IL: Center for Connected Learning and Computer-Based Modeling. See http://ccl.northwestern.edu/netlogo/.

[52] Cockrell C , An G . 2021 Comparative computational modeling of the bat and human immune response to viral infection with the comparative biology immune agent based model. Viruses **13** , 1999–4915. (10.3390/v13081620) 34452484 PMC8402910

[53] Laubenbacher R *et al* . 2024 Toward mechanistic medical digital twins: some use cases in immunology. Front. Digit. Health **6** , 1349595. (10.3389/fdgth.2024.1349595) 38515550 PMC10955144

[54] Laubenbacher R , Mehrad B , Shmulevich I , Trayanova N . 2024 Digital twins in medicine. Nat. Comput. Sci. **4** , 184–191. (10.1038/s43588-024-00607-6) 38532133 PMC11102043

[55] Lynch P . 2008 The origins of computer weather prediction and climate modeling. J. Comput. Phys. **227** , 3431–3444. (10.1016/j.jcp.2007.02.034)

[56] Leutbecher M , Palmer TN . 2008 Ensemble forecasting. J. Comput. Phys. **227** , 3515–3539. (10.1016/j.jcp.2007.02.014)

[57] Zhang H , Pu Z . 2010 Beating the uncertainties: ensemble forecasting and ensemble-based data assimilation in modern numerical weather prediction. Adv. Meteorol. **2010** , 1–10. (10.1155/2010/432160)

[58] Slingo J , Palmer T . 2011 Uncertainty in weather and climate prediction. Phil. Trans. R. Soc. A **369** , 4751–4767. (10.1098/rsta.2011.0161) 22042896 PMC3270390

[59] Bauer P , Thorpe A , Brunet G . 2015 The quiet revolution of numerical weather prediction. Nature **525** , 47–55. (10.1038/nature14956) 26333465

[60] Buehner M , McTaggart-Cowan R , Heilliette S . 2017 An ensemble Kalman filter for numerical weather prediction based on variational data assimilation: VarEnKF. Mon. Weather Rev. **145** , 617–635. (10.1175/mwr-d-16-0106.1)

[61] Alley R , Emanuel K , Zhang F . 2019 Advances in weather prediction. Science **363** , 342–344. (10.1126/science.aav7274) 30679358

[62] Kevrekidis IG , Samaey G . 2009 Equation-free multiscale computation: algorithms and applications. Annu. Rev. Phys. Chem. **60** , 321–344. (10.1146/annurev.physchem.59.032607.093610) 19335220

[63] Gear CW , Hyman JM , Kevrekidid PG , Kevrekidis IG , Runborg O , Theodoropoulos C . 2003 Equation-free, coarse-grained multiscale computation: enabling mocroscopic simulators to perform system-level analysis. Commun. Math. Sci. **1** , 715–762. (10.4310/cms.2003.v1.n4.a5)

[64] Kevrekidis IG , Gear CW , Hummer G . 2004 Equation‐free: the computer‐aided analysis of complex multiscale systems. AIChE J. **50** , 1346–1355. (10.1002/aic.10106)

[65] Erban R , Kevrekidis IG , Othmer HG . 2006 An equation-free computational approach for extracting population-level behavior from individual-based models of biological dispersal. Phys. D **215** , 1–24. (10.1016/j.physd.2006.01.008)

[66] Kalman RE . 1960 A new approach to linear filtering and prediction problems. J. Basic Eng. **82** , 35–45. (10.1115/1.3662552)

[67] Särkkä S . 2013 Bayesian filtering and smoothing. Cambridge, MA: Cambridge University Press.

[68] Humpherys J , Redd P , West J . 2012 A fresh look at the Kalman filter. SIAM Rev. **54** , 801–823. (10.1137/100799666)

[69] McElhoe BA . 1966 An assessment of the navigation and course corrections for a manned flyby of Mars or Venus. IEEE Trans. Aerosp. Electron. Syst. **AES-2** , 613–623. (10.1109/taes.1966.4501892)

[70] MC Gee LA , Schmidt SF , Smith GL . 1962 Application of statistical filter theory to the optimal estimation of position and velocity on board a circumlunar vehicle. Hampton VA: NASA Langley Research Center.

[71] Katzfuss M , Stroud JR , Wikle CK . 2016 Understanding the ensemble Kalman filter. Am. Stat. **70** , 350–357. (10.1080/00031305.2016.1141709)

[72] Julier SJ , Uhlmann JK . 2004 Unscented filtering and nonlinear estimation. Proc. IEEE **92** , 401–422. (10.1109/jproc.2003.823141)

[73] Shannon CE . 1948 A mathematical theory of communication. Bell Syst. Tech. J. **27** , 379–423. (10.1002/j.1538-7305.1948.tb01338.x)

[74] Aggarwal C . 2024 Probability and statistics for machine learning: a textbook. Cham, Switzerland: Springer. (10.1007/978-3-031-53282-5)

[75] Bartocci E , Lió P . 2016 Computational modeling, formal analysis, and tools for systems biology. PLoS Comput. Biol. **12** , e1004591. (10.1371/journal.pcbi.1004591) 26795950 PMC4721667

[76] Cruz DA , Kemp ML . 2022 Hybrid computational modeling methods for systems biology. Prog. Biomed. Eng. **4** , 012002. (10.1088/2516-1091/ac2cdf) 41287231

[77] Machado D , Costa RS , Rocha M , Ferreira EC , Tidor B , Rocha I . 2011 Modeling formalisms in systems biology. AMB Express **1** , 45. (10.1186/2191-0855-1-45) 22141422 PMC3285092

[78] Wilensky U , Rand W . 2015 An introduction to agent-based modeling: modeling natural, social, and engineered complex systems with netlogo. Cambridge, MA: MIT Press.

[79] Wilensky U , Reisman K . 2006 Thinking like a wolf, a sheep, or a firefly: learning biology through constructing and testing computational theories—an embodied modeling approach. Cogn. Instr. **24** , 171–209. (10.1207/s1532690xci2402_1)

[80] Wilensky U . 1999 NetLogo. Northwestern University, Evanston, IL: Center for Connected Learning and Computer-Based Modeling. See http://ccl.northwestern.edu/netlogo/.

[81] Knapp AC . wolf-sheep-grass. Github (MIT License). See https://github.com/knappa/wolf-sheep-grass.

[82] Ranger RH . 1931 Facsimile system. US Patent no. US1790723A.

[83] Floyd RW . 1976 An adaptive algorithm for spatial gray-scale. Proc. Soc. Inf. Disp. **17** , 75–77.

[84] Villani C . 2009 In The Wasserstein distances, pp. 93–111. Berlin, Germany: Springer. (10.1007/978-3-540-71050-9_6)

[85] Uhlmann J , Julier SJ . 2022 Gaussianity and the Kalman filter: a simple yet complicated relationship. J. De Cienc. E Ing. **14** , 21–26. (10.46571/jci.2022.1.2)

[86] Killilea DW , Kuypers FA , Larkin SK , Schultz K . 2022 Blood draw site and analytic device influence hemoglobin measurements. PLoS One **17** , e0278350. (10.1371/journal.pone.0278350) 36449486 PMC9710840

[87] Cable RG *et al* . 2012 The difference between fingerstick and venous hemoglobin and hematocrit varies by sex and iron stores. Transfusion **52** , 1031–1040. (10.1111/j.1537-2995.2011.03389.x) 22014071 PMC3623687

[88] Lock JP , Szuts EZ , Malomo KJ , Anagnostopoulos A . 2002 Whole-blood glucose testing at alternate sites. Diabetes Care **25** , 337–341. (10.2337/diacare.25.2.337) 11815506

[89] Ribeiro HA , Vieira LS , Scindia Y , Adhikari B , Wheeler M , Knapp A , Schroeder W , Mehrad B , Laubenbacher R . 2022 Multi-scale mechanistic modelling of the host defence in invasive aspergillosis reveals leucocyte activation and iron acquisition as drivers of infection outcome. J. R. Soc. Interface **19** , 20210806. (10.1098/rsif.2021.0806) 35414216 PMC9006013

[90] Baker RE , Simpson MJ . 2010 Correcting mean-field approximations for birth-death-movement processes. Phys. Rev. E **82** , 041905. (10.1103/physreve.82.041905) 21230311

[91] Chen C . 1999 Linear system theory and design. Oxford, UK: Oxford University Press.

[92] Brunton S , Kutz J . 2022 Data-driven science and engineering: machine learning, dynamical systems, and control, 2nd edn. Cambridge, MA: Cambridge University Press.

[93] Knapp A , Cruz D . 2025 enkf-on-abms. Zenodo. (10.5281/zenodo.15319066)

[94] LaboratoryForSystemsMedicine . 2024 enkf-on-abms. See https://github.com/LaboratoryForSystemsMedicine/enkf-on-abms.

[95] Knapp A , Cruz DA , Mehrad B , Laubenbacher RC . 2025 Supplementary material from: Personalizing computational models to construct medical digital twins. Figshare. (10.6084/m9.figshare.c.7828981) PMC12212996 40592464

